# Molecular Biogeography: Towards an Integrated Framework for Conserving Pan-African Biodiversity

**DOI:** 10.1371/journal.pone.0000454

**Published:** 2007-05-23

**Authors:** Yoshan Moodley, Michael W. Bruford

**Affiliations:** School of Biosciences, Cardiff University, Cardiff, United Kingdom; Freie Universitaet Berlin, Germany

## Abstract

**Background:**

Biogeographic models partition ecologically similar species assemblages into discrete ecoregions. However, the history, relationship and interactions between these regions and their assemblages have rarely been explored.

**Methodology/Principal Findings:**

Here we develop a taxon-based approach that explicitly utilises molecular information to compare ecoregion history and status, which we exemplify using a continentally distributed mammalian species: the African bushbuck (*Tragelaphus scriptus*). We reveal unprecedented levels of genetic diversity and structure in this species and show that ecoregion biogeographic history better explains the distribution of molecular variation than phenotypic similarity or geography. We extend these data to explore ecoregion connectivity, identify core habitats and infer ecological affinities from them.

**Conclusions/Significance:**

This analysis defines 28 key biogeographic regions for sub-Saharan Africa, and provides a valuable framework for the incorporation of genetic and biogeographic information into a more widely applicable model for the conservation of continental biodiversity.

## Introduction

Continental-scale biogeographic models capture and incorporate the complexity of natural ecosystems by partitioning this variation into provinces or ‘ecoregions’ which can be used as manageable foci for the conservation of biodiversity [1], [2]. The Afrotropical Biogeographic Realm [3] contains one of the highest levels of biodiversity on Earth. This model divides continental Africa into a series of biogeographical provinces on the basis of ecosystematic or biotic features. Biogeographic provinces can be translated loosely into floral [4], [5] and eco-faunal regions [6]. A recent analysis of the Afrotropical Biogeographic Realm [7] accounted for intra-province variation and defined smaller ‘ecoregions’ with greater ecological specificity, such as seasonal floodplains and mangrove swamps. Besides helping to enhance and focus the management of conservation efforts [eg. 8] biogeographic models can hint at recent evolutionary processes [9] that have given rise to the faunal and floral assemblages associated with each ecoregion. However, species assemblages may differ extensively between different ecoregions due to unequal taxonomic effort and scale effects [10] such that only contiguous and/or very similar ecoregions can be readily compared, which hampers objective estimates of alpha and gamma diversity. A further problem is that research has ignored taxa with wide-scale evolutionary histories, including adaptation within a large range of key ecoregions, in favour of specialists, which are often of greater conservation concern and serve as examples of singular threatened ecoregions.

However, a number of potential species are sufficiently widespread and adapted to biogeographic provinces at a continental scale to be potential models. One example in Africa is the bushbuck (*Tragelaphus scriptus*): a mammalian generalist and herbivore that has kept pace with environmental changes by local adaptation to changing habitats, since this sedentary species appears to require only water, cover and the availability of grazing or browse [11]. As a consequence, *T. scriptus* is Africa's most widely distributed ungulate, occurring in every country in sub-Saharan Africa with its range limited only by the lowland closed canopy forests of the Central Congo Basin, deserts and highly xeric shrublands. Hence, bushbuck remain common in Equatorial and Guinean lowland forests [12] while inhabiting montane forests across the continent at elevations up to 4000 m [13] and even penetrating into the xeric Sahel and Somali zones along seasonal watercourses [11], [14]. The bushbuck inhabits 17 of the 19 terrestrial Sub-Saharan biogeographical provinces in Udvardy's [3] model and 62 of the 91 ecoregions in the extended Olson *et al.* [7] model. This equates to approximately 73% of the total land area of Sub-Saharan Africa.

Local adaptation across this vast and heterogeneous range has resulted in marked geographic variation in body and horn size, coat length and pattern, colouration and sexual dimorphism [14], [15]. Over 40 subspecies have been described but systematic studies indicate that between 24 [16], [17] and six [15] distinct forms may exist. The structure of mitochondrial genetic diversity in grassland [18]–[20], forest [21] and arid-adapted African species [22], [23] has previously been analysed in a phylogeographic context. These studies provide essential information on evolutionary history with respect to the paleohistories of discrete, ecologically homogeneous regions, but do not reveal how these distinct ecological regions are linked. The bushbuck provides an opportunity for establishing such links due to its unsurpassed phenotypic diversity, comprising forest, savanna, woodland, montane and arid-adapted forms.

Using this model taxon approach to continental biogeography provides a quantitative framework for the integration of traditional approaches with ecological data, because it partitions diversity into regionally meaningful components and links biogeographic ecoregions with the evolutionary history of ubiquitous species. Novel inferences about ecoregional connectivity, core habitats, ecological affinities and adaptation then become possible and regions of core evolutionary and ecological importance can be identified. This approach requires extensive sampling to validate the resulting inferences. In this study we assessed the evolutionary history of the bushbuck from an unprecedented, continent-wide sample using mitochondrial DNA control region sequences and a complementary data set for the cytochrome *b* gene. We analyse genetic structure in the context of biogeographic history, phenotype and geography, and using a new approach to combine genetic and ecological data, we elaborated a taxon-linked model for pan-African molecular biogeography.

## Results

We examined 516 bp of sequence from the 5′ end of the *Tragelaphus scriptus* mitochondrial control region (CR) in 485 specimens covering the entire species range and accounting for all known phenotypic variation (Table S1). We also examined 556 bp of the mitochondrial cytochrome *b* gene (cyt *b*) from a sub-sample of 161 specimens. 259 sites (50.2%) were polymorphic for the CR and 159 sites (28.6%) for cyt *b*, yielding 320 and 90 unique haplotypes, respectively. Nucleotide diversity (*π*) for both sequences was very high (*π*CR: 11.7%, *π*cyt *b*: 7.4%).

### Genetic Structure

**Figure 1:**
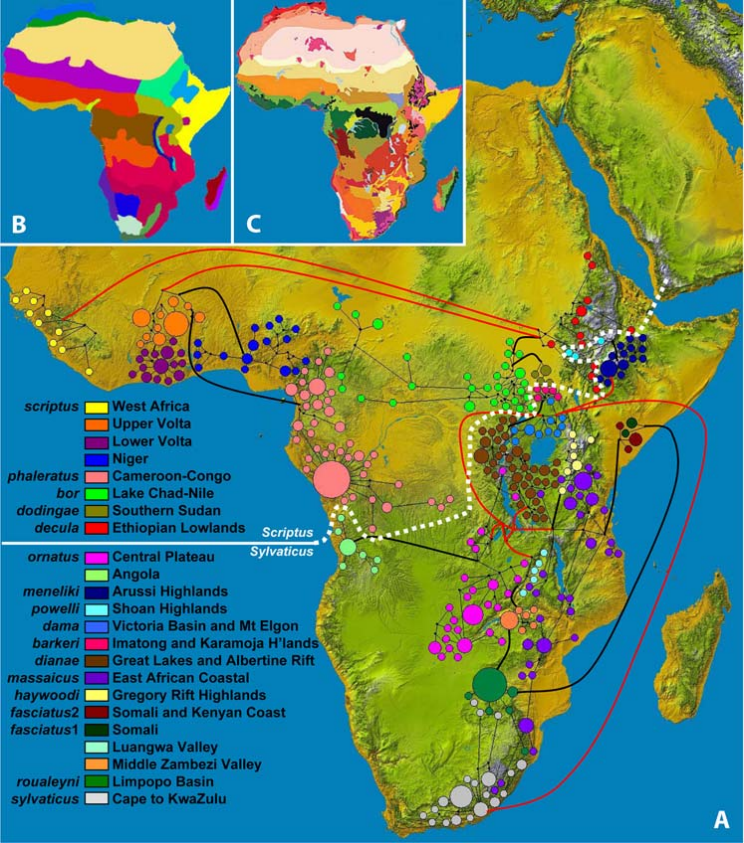
Pan-African relationships among 23 bushbuck haplogroups in relation to toplogy and habitat heterogeneity. Part A. An unrooted median joining network of 485 control region sequences, overlaid onto a topographical map of Africa. 320 haplotypes are arranged according to original sampling location, thus describing the approximate geographic range of each terminal haplogroup. The size of each circle is scaled to haplotype frequency. A dashed white line separates *Scriptus* and *Sylvaticus* haplotypes. Relationships between intermediate-level haplogroups (thick red links) were unresolved by CR data and were inferred from a cytochrome *b* network (data not shown). Thick black links join terminal haplogroups nested within intermediate haplogroups and thin black links join individual haplotypes within terminal haplogroups. Inset panels B and C show the comparitive distribution of biogeographic provinces [3] and ecoregions [7] respectively.

**Figure 2:**
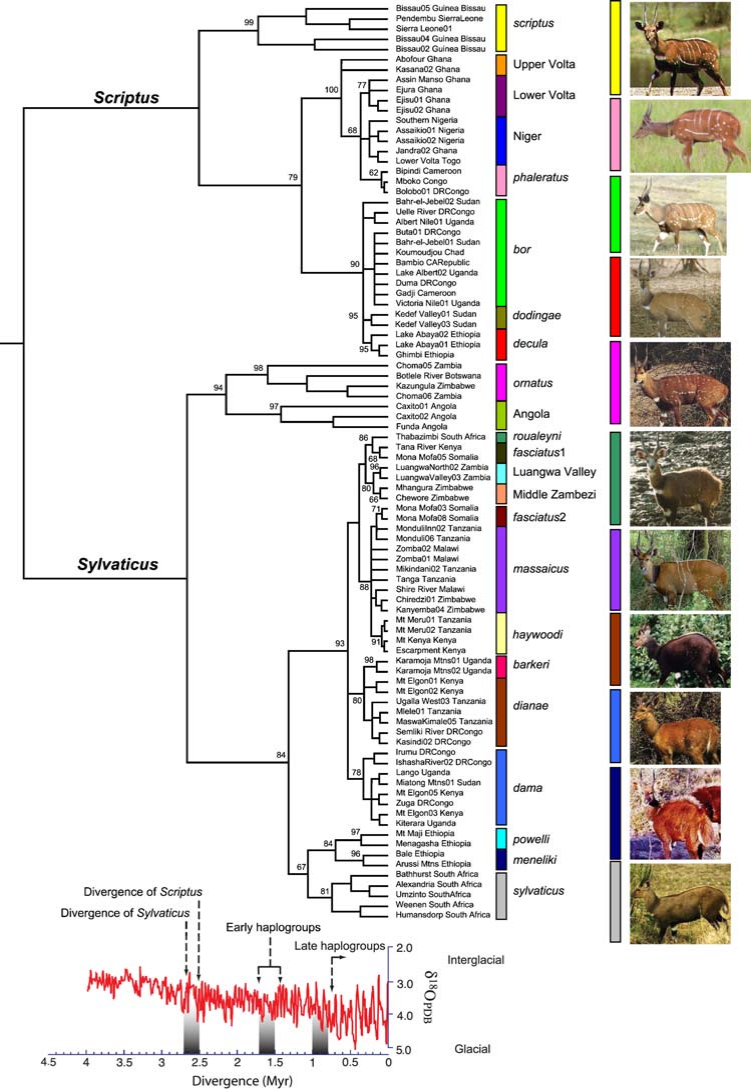
Linearised phylogenetic tree for 90 cytochrome *b* haplotypes obtained by maximum likelihood assuming an HKY model for nucleotide substitution. Nodal support for haplogroups is displayed and nodes with less than 50% support were collapsed. Haplogroups are determined by monophyly and distribution. Haplogroups are colour coded as in Figure 1. The picture panel shows the general phenotype of each intermediate-level haplogroup. The *decula* terminal haplogroup is depicted to show the loss of patterning in this subspecies. The bottom panel displays the timing of evolutionary events with equivalent changes in the global temperature (as measured by changes in the oxygen isotope 18) adapted from [55].

**Figure 3:**
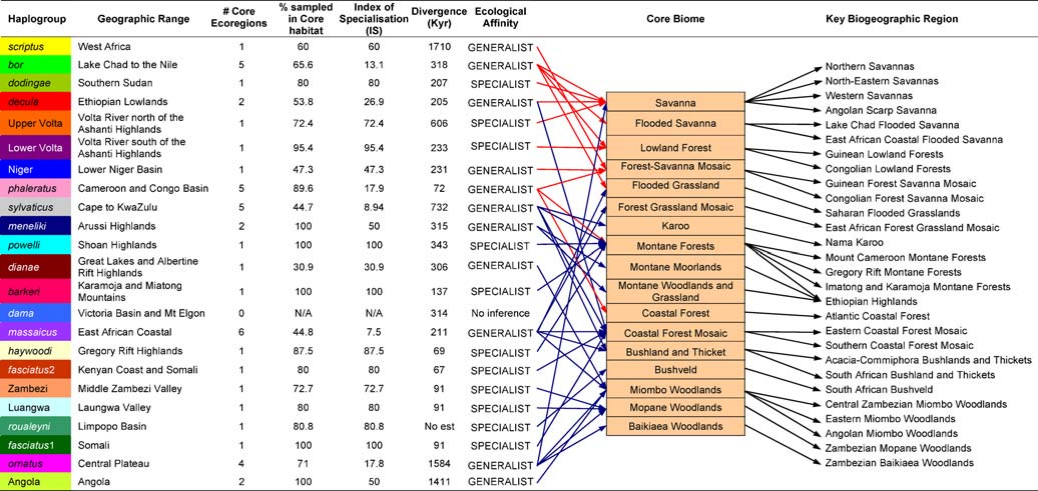
Ecoregional habitation of 23 bushbuck haplogroups, the inference of ecological affinities and the identification of key biogeographic regions as defined by molecular data. Ecological affinity was inferred from a haplogroup's index of specialisation (IS); Arrows (red for the *Scriptus* group and blue for the *Sylvaticus* group) summarise the core ecoregions of each haplogroup into their appropriate core biomes (shaded), which were then translated into key biogeographic regions by the inclusion of geography. Haplogroups are colour-coded as in Figure 1.

Genetic structure was assessed by a median-joining network for CR haplotypes (Fig. 1) and by maximum likelihood (ML) for the cyt *b* data set (Fig. 2). The results show that all genetic variation was partitioned into 2 basal haplogroups which divide sub-Saharan Africa roughly along the axis of the Rift Valley into Western-Northern and Eastern-Southern sectors (Fig. 1); hereafter referred to as the *Scriptus* [Pallas 1766] and *Sylvaticus* [Sparrman 1780] groups respectively, in accordance with the earliest described taxon from each group. Both groups possessed high nucleotide diversity (*Sylvaticus*: *π*CR 6.2%, *π*cyt *b* 3.5%; *Scriptus*: *π*CR 5.2%, *π*cyt *b* 3.6%). Additional substructure was extremely high, with high bootstrap support (Fig. 2) for 23 terminal haplogroups with distinct geographic ranges (Fig. 1). The cyt *b* data resolved the branching order of intermediate-level nodes more efficiently, partitioning the *Scriptus* group into three intermediate and eight terminal level haplogroups and the *Sylvaticus* group into seven and 15 intermediate and terminal haplogroups, respectively (Fig. 2, Table S2). All 23 terminal haplogroups were monophyletic, however four (labelled ‘*bor*’, ‘*phaleratus*’, ‘*dianae*’ and ‘*massaicus*’) were not reciprocally monophyletic relative to haplogroups nested within them. The division of the major *Scriptus* and *Sylvaticus* groups (picture panel, Fig. 2) corresponded well with phenotype, showing marked (*scriptus*) and moderate (*ornatus*) white patterning in the phylogenetically basal haplogroups of both groups and a general loss of patterning in more derived forms. Evolution of darker, thicker coats and larger size within the *Sylvaticus* group appears to have arisen independently on the montane inselbergs of East Africa. Due to the high geographic structure in the data (Fig. 1), discrete population units could be inferred directly from the terminal haplogroups (Fig. 2). Previously described subspecies provide convenient (although not necessarily biologically meaningful) labels for most terminal haplogroups, however in six instances the molecular data support non-taxonomic groupings; namely in Angola, the Middle Zambezi Valley, the Luangwa Valley, Upper Volta, Lower Volta and Niger (Table S2). A relaxed Bayesian molecular clock [24] dated the coalescence of the cyt *b* lineages to the late Neogene, 4.8 Mya (95% CI: 3.9–6.5 Mya). While diversification into the major *Sylvaticus* and *Scriptus* groups was dated to the late Pliocene at 2.7 and 2.5 Mya respectively (95% CL: 3.0–2.0 Mya), during a time of considerable faunal turnover in Africa [25], [26], individual haplogroups date to the Pleistocene between 1.7 and 0.067 Mya (Fig. 3).

### Phenotype models

Partitioning all sequences into their respective terminal haplogroups showed that only 9% (AMOVA) to 14% (multivariate matrix regression, MMR) of the variation in the CR data occurs at the within-population level (Table 1). Of the six taxonomic hypotheses that attempt to partition bushbuck on the basis of phenotype, the 24-group Lydekker [16] model and the 10-group Grubb-Best [15], [27] combined model provided the best congruence between genetic divergence and phenotype. Despite this, both models still only explained 64% and 63% of the variation in the data respectively (Table 1). The better performance of Lydekker's older model challenges the wisdom of later attempts to obtain a manageable overview of bushbuck variation by synonymising phenotypically variable subspecies on the basis of geographical proximity. Even the one-group subspecies comparison, which provides a measure of overall structure in the data relative to subspecies definitions, showed that between 29% (AMOVA) and 31% (MMR) of the molecular variance in the data was found within-populations, highlighting a poor molecular correlation with the present taxonomic designations in bushbuck.

**Table 1:** Model testing by analyses of molecular variance (AMOVA) and multivariate matrix regressions (MMR).

			AMOVA	Multivariate matrix regression		
	Model	#Groups	*F*_ST_ or *F*_CT_	Marginal	Conditional	Sequential
Phenotype	Lydekker (16)	24	0.6425***	—	—	—
	Allen (17)	24	0.4371*	—	—	—
	Best (38)	9	0.3893***	—	—	—
	Haltenorth (39)	21	0.4424***	—	—	—
	Grubb (15)	6	0.5700***	—	—	—
	Grubb-Best (15, 38)	10	0.6281***	—	—	—
	All subspecies	1	0.7062***	0.6866***	0.4458***	S, G: 0.0167***
Biogeography	Udvardy (3)	1	0.5920***	0.4793***	0.2866***	B, G: 0.0600***
	Olson *et al.* (7)	1	0.7568***	0.7688***	0.5259***	B, G: 0.0072**
	Refined Ecoregional Model	1	0.8205***	0.8036***	0.5545***	B, G: 0.0030^NS^
Molecular	Terminal Haplogroups	1	0.9122***	0.8589***	0.6134***	M, G: 0.0046**
Geography	Coordinates	1	—	0.2500***	—	Lt, Lg: 0.0408***

The test statistic reported is the proportion of variance in the data that was accounted for by each model. For AMOVA this may be the proportion of variance among populations (*F*_ST_, in bold case, where #groups = 1) or among groups (*F*_CT_, normal case, where #groups>1). Conditional MMR tests assess the proportion of marginal variation remaining after geography is taken into account. Sequential MMR tests fit the best model to the data and the statistic reported is that proportion of the remaining variation that is attributable to the second model. Models (P, phenotype; B, biogeography; G, geography; M, molecular; Lt, latitude; and Lg, longitude) are listed in the sequential order in which they were fitted to the regression. Significance was denoted *** (p<0.001), ** (p<0.01), * (p<0.05) or NS (not significant) after 10000 (AMOVA) or 9999 (MMR) permutations.

### Biogeographic models

Of the two biogeographic models tested, the Udvardy model accounted for 59% (AMOVA) to 48% (MMR) of CR variation in 17 biogeographic provinces (Table 1). However, the Olson model which includes 62 Afrotropical ecoregions described 76–77% of the variation in the CR data, significantly greater even than the 69–71% accounted by taxonomic designation. Of the 50 ecoregions in our data set, 27 are inhabited by only a single haplogroup (Table S3). The remaining 23 ecoregions were inhabited by individuals belonging to more than one haplogroup and hence comprises the 15% difference in explanatory molecular variance between the Olson model and the CR data. These 23 ‘shared ecoregions’ could be partitioned into those where haplogroup ranges were mutually exclusive and those where they overlapped. The six shared ecoregions with mutually exclusive haplogroup distributions occurred as a result of a) broadly defined ecoregions where haplogroups are separated either by distance (Guinean forest-savanna mosaic) or unfavourable habitat (Sahelian Acacia savanna, East Sudanian savanna); b) separation by geomorphological barriers such as escarpments, river or rift valleys (Zambezian and Mopane woodlands, Central Zambezian Miombo woodlands) and c) comprising of groupings of distinct insular ecosystems (East African montane forests). These results indicate that even the most detailed biogeographic models available have limitations when including isolation by distance, physical barriers to gene flow, and habitat heterogeneity. We therefore refined the Olson model by partitioning the above six ecoregions with mutually exclusive haplogroup distributions into 14 sub-ecoregions (cf. Table S3) where haplogroup ranges are not mutually exclusive. This revised ecoregional model divides sub-Saharan Africa into 58 ecoregions and accounts for 80–82% of the variation in the CR data set (Table 1). Nevertheless, a further 22 shared ecoregions (five of which were newly defined, Table S3) are co-inhabited by more than one haplogroup and these collectively account for the 6–9% difference in variance between the refined ecoregional model and the molecular (terminal haplogroup) model. We did not attempt to further revise the model, given that the bushbuck is an adaptable generalist, and individuals may often range outside of the limits of their core habitat.

### Geography

Geographic distance accounted for 25% of the variation in the CR data set, only 4% of which was attributed to longitude. That latitudinal variation comprises the majority of the geographical variance may not be surprising, given the distribution of sampling locations from as far as 15.7°N to 34°S (Table S1), and the variety of habitats occurring within this range. Nevertheless, both biogeography and phenotype still explained significantly higher levels of variation over and above the conditional influence of geography (Table 1). Furthermore, sequential multivariate regressions always ranked geography as less important than any of the other models tested. Except in the case of the Udvardy model, geographic distance accounted for very little (<2%) of the molecular variation that remained once the best model was fitted to the regression, implying that phenotypic and the more detailed biogeographic models already take most of the geographic variation into account.

### Core habitats

We defined the core habitats of each haplogroup on the basis of the distribution of its members. Ecoregions were considered part of a haplogroup's core habitat if they were inhabited exclusively or if they contained more than 50% of individuals in a terminal haplogroup. Implementing these criteria, 44 of the 58 ecoregions in the model were defined as core ecoregions to the 23 terminal haplogroups (Table S3). According to our redefined ecoregional model, haplogroup presence ranged from the habitation of a single core ecoregion to occurrence in up to four (*ornatus*), five (*bor, phaleratus* and *sylvaticus*) and maximally six (*massaicus*; Fig. 3). This core habitat concept allowed the molecular inference of haplogroup ecological affinity and formed the basis for the definition of core biomes and regions of key biogeographic importance.

## Discussion

### Origins and Evolution

The bushbuck is the most widespread and taxonomically diverse ungulate on the African continent and we have shown that all molecular variation in our dataset may be partitioned into two divergent lineages. Fossil remains of *T. scriptus* are known from several sites in eastern and southern Africa, but its appearances in the fossil record, from locations in Kenya [28], [29] and Ethiopia [30] as early as 3.9 Mya, predate the diversification of *Sylvaticus* and *Scriptus* lineages and suggest north-east Africa as the centre of origin for this species. While this region is open and xeric today, it was thickly forested until the late Pliocene [31], [32], [33]. The presence of common phenotypic characteristics of *scriptus* and *ornatus* - the two most basal haplogroups within the *Scriptus* and *Sylvaticus* groups (picture panel, Fig. 2), the species' preference for cover and the fact that all juvenile bushbuck are patterned [11], [13], suggests that ancestral bushbuck were also strikingly patterned and adapted to the dappled light of forest habitat.

A global climatic shift approximately 2.8 Mya (see lower panel Fig. 2) is thought to have resulted in a major increase in grassland habitats and, consequently, a dramatic change (or turnover pulse; [25]) in species composition, most notably in north-east Africa [34], [35]. The divergence of the *Sylvaticus* (2.7 Mya) and *Scriptus* (2.5 Mya) lineages coincides with this paeleoclimatic event, and the resulting loss of patterning in derived bushbuck haplogroups is indicative of the expansion and diversification of ancestral populations into more open habitats. However, paeleoclimatic events and ensuing diversification/specialisation do not explain the independent evolutionary trajectories of the *Sylvaticus* and *Scriptus* lineages, as extant phenotypic diversity and ecoregional habitation (Table S3) indicates that ecological specialisation is derived, and haplogroups within the two lineages are still capable of exploiting both forested and open niches. The major tectonic uplift events along the Gregory and Albertine Rifts also date to the late Pliocene (3–2 Mya), [31], [32] and the extant distributions of *Sylvaticus* and *Scriptus* groups on either side of the Rift Valley (Fig. 1) suggest allopatric separation of the two lineages, initially on the basis of geomorphology. Thereafter, diversification can be shown to be influenced primarily by paeleoclimate. The dominance of the ancestral coat patterning within the *Scriptus* lineage implies a longer association with forested habitats and reflects differences in paeleoclimate on either side of the Rift Valley. Nevertheless the diversification of the first bushbuck haplogroups (*scriptus, ornatus* and Angola) between 1.7 and 1.4 Mya (Fig. 2), is hypothesised to have been in response the expansion of grasslands during the glacial period of the early Pleistocene, around 1.7 Mya [36]. The phenotypically similar, temperate-adapted haplogroups of the Ethiopian Highlands (*meneliki* and *powelli*) and the Cape (*sylvaticus*) form a sister clade, that may have been more dominant during colder periods, but now only persist in these temperate zone refuges at opposite ends of the continent. All other diversification events occurred less than 800 Kya, after the periodicity of glacial cycles changed from 41 kyr to 100 kyr (lower panel, Fig. 2), producing prolonged periods of cooling in Africa. The late Pleistocene (<400 Kyr) was especially important for divergence in many large African mammal species [36] and 18 of the 23 bushbuck haplogroups defined in this study are estimated to have diversified during this period. While it may not be possible to accurately reconstruct the timing of events at this timescale, it is interesting to note the convergence of phenotypically similar montane forms in East Africa during glacial maxima 137 Kya (*barkeri*) and 69 Kya (*haywoodi*), as well as the contraction of semi-arid-adapted populations (*fasciatus*1, Zambezi Valley and Luangwa Valley) to drier refugia during the glacial minimum, 125–80 Kya.

### Model testing

Despite a number of previous attempts to partition the vast array of phenotypic variation within this species [11], [13]–[17], [27], [37], [38], inclusion of all described phenotypes account for only 71% (AMOVA) and 69% (MMR) of the variance in the CR data (Table 1), and more variation can be explained using the molecular data alone. The relative failure of the phenotype models is largely due to the inability of taxonomists to recognise phenotypic variation within the subspecies *scriptus* (formerly ranging from Senegal to Congo) and *ornatus* (formerly ranging in South-Central Africa) due to consideration of only a handful of historically popular phenotypic characteristics such as coat colour, patterning, horn and body size and hair length. The use of more rigorous taxonomic methods such as geometric morphometrics may increase phenotypic resolution of these traditionally accepted subspecies. The present study (Fig. 1) partitions the historic *scriptus* into West Africa (*scriptus*), Upper Volta, Lower Volta, Niger, and Cameroon-Congo (*phaleratus*) haplogroups and historic *ornatus* into Central Plateau (*ornatus*), Angola, Luangwa Valley, Middle Zambezi Valley and East African Coastal (*massaicus*) haplogroups. Biogeographic ecoregions [7] provided a more solid foundation for the description of species history, especially after the incorporation of potential barriers to dispersal such as unfavourable habitat, distance and altitudinal gradients (Table 1). This strong association between genetic structure and biogeography strengthens our choice of the bushbuck as a model taxon for the elucidation of biogeographical processes in the Afrotropical Realm. Molecular biogeography - defining genetic structure in terms of a biogeographic model - allows the investigation of a number of ecological and evolutionary processes that would otherwise be intractable. These are discussed below.

### Connectivity between Ecoregions and the inference of Ecological affinities

**Figure 4:**
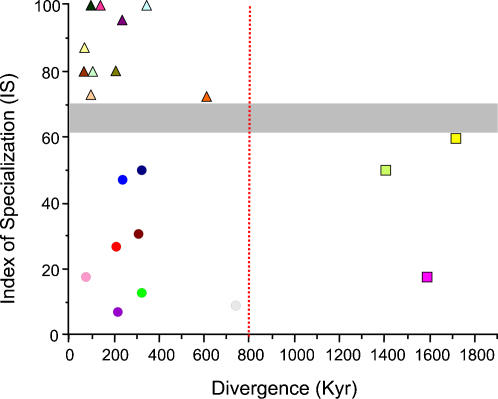
The evolution of specialization through time in bushbuck. The shaded grey area marks the threshold distinguishing specialist from generalist haplogroups and the dashed red line denotes the onset of major diversification events. Boxes denote early generalists; circles, late generalists; and triangles, late specialists. Haplogroups are colour coded as in Figure 1.

The number of sampled ecoregions (58) in our taxon-linked ecoregional model is more than twice the number of terminal haplogroups defined by mitochondrial DNA, which suggests considerable connectivity between ecoregions. Since only between 6 and 9% of the genetic variation in the CR data is found within haplogroups, we assume that adjacent ecoregions that are inhabited by a single haplogroup are maximally connected. This assumption only holds true for ecoregions that constitute a haplogroup's core habitat since ecoregions on the outer limits of a haplogroup's range are more likely to experience unidirectional gene flow. The nature of the ecoregions being connected by a single haplogroup therefore provides a measure of a haplogroup's ecological affinity. If the core habitat of a haplogroup comprises a single ecoregion, (Fig. 3) then that haplogroup is more likely to be an ecological specialist, whereas generalists would be expected to inhabit more than one ecoregion. The proportion of each haplogroup that was sampled within its core habitat provides a second, complementary measure of ecological affinity as this value will be lower for generalists. Dividing the proportion of each haplogroup sampled in its core habitat by the number of core ecoregions in which that haplogroup was sampled (columns 3–5, Fig. 3) describes an index of specialisation (IS). This index accounts for those haplogroups that inhabit a single core habitat, but are present at lower frequency in diverse secondary habitats (eg. *scriptus*, Niger and *dianae*). When IS was plotted against haplogroup divergence time, a continuum from generalist to specialist haplogroups became apparent, with a transition in ecological affinity evident between IS = 60–70% (shaded grey area, Fig. 4). Furthermore, three separate groupings are present in Figure 4. Early generalists *scriptus*, *ornatus* and Angola comprise the most phylogenetically basal (see Fig. 2) of the bushbuck haplogroups, and strongly suggest a generalist ancestral state for this species. An increase in the length and severity of glacial cycles led to an increase in the number of diversification events after 800 Kya. Not all diversification, however, resulted in specialisation: the proliferation of late generalists shows that ubiquitous haplogroups were continually able to exploit diverse habitats from the late Pleistocene to the present. Late specialists are associated with montane inselbergs, semi-arid zones and close canopy forests, and confirm that less ubiquitous haplogroups are derived. The strong association between haplogroups and their core habitats means that early lineages were able to colonise newly created ecoregions, adapt to these ecoregions and diversify within them with little inter-habitat migration. The persistence of these lineages and their close association with habitat to the present day, therefore, hints at the persistence of these ecoregions through the major climatic oscillations of the Pleistocene.

While 44 of the 58 ecoregions defined in this model were found to be core to the 23 haplogroups as a whole, the proportion of each haplogroup sampled in core habitat varied from as little as 30.9% to 100% (Fig. 3). In order to define ecologically meaningful biogeographic regions, we integrated the core habitat concept into our taxon-linked model. This translated to 17 core biomes, with the haplogroup biome diversity graphically described by linking each terminal haplogroup to a biome if it constituted part of its core habitat (blue and red arrows, Fig. 3). The *Scriptus* and *Sylvaticus* groups were colour coded for ease of inference. The *Scriptus* forms inhabit lowland forest, savanna, and coastal forest exclusively while *Sylvaticus* forms occur exclusively in a much wider range of biomes. Of the three biomes that are shared between the major groups, montane forests were the most widely inhabited by both forms, further demonstrating the adaptive nature of this species to changes in paeleoclimate and geology. It should be noted that this does not imply that *Scriptus* and *Sylvaticus* groups are ecologically distinct, but rather reflects the different ecological characteristics of habitats in the Western-Northern and Eastern-Southern halves of Africa.

### Key Biogeographic Regions

The strong relationship between haplotype structure in the bushbuck and Afrotropical biogeographic structure forms the basis for a model-taxon approach, complementary to, but different from, classical species-assemblage biogeography and traditional phylogeography, because it takes ecological factors into account in testable hypotheses. Implicit in this approach is the need to include several key organisms of different dispersal ability in any similar assessment and as many molecular markers (including those known to be under diversifying selection) as possible. An essential final development to our model is the addition of a geographical component to each of the core biomes that identifies centres of ecological importance. We thus proposed 28 key biogeographic regions (Fig. 3) as defined by the genetic associations of our model taxon in ecologically heterogeneous habitats. This model differs from previously defined species assemblage models in that it links ecological regions on the basis of genetic similarity and in doing so provides a quantitative framework for the inclusion of evolutionary processes in the description of biodiversity.

With respect to our model taxon, we suggest that these 28 key biogeographic regions summarise the core habitats essential for maintaining continental-scale processes within the bushbuck complex. However, the ubiquity and ecological diversity of this species emphasises the potentially widespread applicability of this model to forest, savanna, montane, woodland and arid adapted species, as well as to other African generalists and means that a molecular biogeographical approach to conservation, using the 28 key biogeographic regions of our model as foci for conservation, will invariably conserve areas of core importance to most Afrotropical mammals. A well-chosen set of organisms and markers can potentially extend the applicability of this approach to a fuller ecoregion analysis.

## Methods

### Samples and molecular methods

485 skin or tissue specimens were collected from museum collections, hunters and taxidermists covering 239 locations in 27 sub-Saharan countries and included 33 putative subspecies (Table S1). Genomic DNA was isolated from ≤500 mg (dry weight) of source material by SDS-proteinase K digestion and phenol-chloroform extraction [39] in an isolated laboratory. Primers MT4 [40] and BT16168H [41] reliably amplify a 400–500 bp non-nuclear 5′ fragment of the mitochondrial control region in African bovids [22], [41], [42]. Control region PCR was carried out on 50 ng DNA in a total reaction volume of 25 µl containing 0.2 mM of each primer, 3.0 mM MgCl_2_, 0.5 mM DNTPs, 1 U Taq DNA polymerase (Invitrogen) and 1× PCR buffer. Cycling conditions were: initial denaturation for 5 min at 95°C; 35 cycles of denaturation for 30 s at 95°C, annealing for 30 s at 58°C and extension for 1 min at 72°C; followed by a final extension phase for 10 min at 72°C. A 556 bp fragment of the cytochrome (cyt) *b* gene was amplified with primers L15162 [43] and H15761 [C. Fernandes, pers. comm.] in 161 samples in order to confirm haplogroups and for the estimation of haplogroup divergence times. The cyt *b* PCR protocol was as above except that reactions contained 0.4 mM of each primer and primer annealing was for 30 s at 55°C. PCR products were purified by digestion with 5 U/µl Exonuclease I and 0.5 U/µl Shrimp Alkaline Phosphatase (Amersham) for 60 min at 37°C, followed by denaturation at 80°C for 15 min. Direct sequencing in both directions was carried out using the BigDye Terminator Kit (Applied Biosystems) and sequencing products were analysed with an ABI 3100 sequencer. Sequences were assembled using Sequencher 4.1.2 (Gene Codes). The 646 sequences have Genbank accession numbers EF138117-EF138601 (CR) and EF137956-EF138116 (cyt *b*).

### Genetic structure

The relationships among haplotypes in the CR data set were visualised in a median joining network [using NETWORK 4.1, 44; Fig. 1]. CR haplotypes were assigned to monophyletic haplogroups (Table S2). The diversity of the cyt *b* data set was examined by a maximum likelihood (ML) phylogenetic analysis (Fig. 2) based on the optimal HKY model of nucleotide substitution [45] as indicated by ModelTest 3.7 [46], using TREEFINDER [47] with empirically determined nucleotide frequencies and transition-transversion ratio. Cyt *b* nodes were dated by a Bayesian relaxed molecular clock as implemented in the PAML 3.14 [48] and MULTIDISTRIBUTE [24], [49]. Four calibration points were invoked: we conservatively estimated the emergence of two main lineages at an upper limit of 3 Mya, and restricted the evolution of Gregory Rift montane isolates to the onset of the last glacial cycle, approximately 110 Kya. We estimated a lower limit of the age of the phylogeny at 3.9 Mya, based on the earliest appearance of *T. scriptus* in the fossil record [30].

### Model testing

Phenotypic and biogeographic model testing of the CR data set (Table 1) was carried out using two methods. Firstly the analysis of molecular variance [AMOVA, 50] framework was implemented, as it allowed the hierarchical partitioning of the data into variance components. For AMOVA analyses, we defined the basic units for each model relative to the phenotypic or biogeographic model being tested. The basic phenotypic unit was the original ‘subspecies’ assignment of each specimen based on taxonomy, which were in turn grouped (or synonymised) according to the classifications of Lydekker [16], Allen [17], Best [38], Haltenorth [39] and Grubb [15]. Grubb's scheme partitioned the phenotypic variation in the species into four broad groups, all of which are represented in East Africa. In order to test Grubb's East African hypothesis over the entire species range, we assumed the Best [38] classification for the individuals in our data set that were sampled outside East Africa. The relative statistical support for these groupings was assessed by the partitioning of variation among groups, among subspecies and within subspecies. To test the biogeographical models of both Udvardy [3] and Olson *et al.* [7] under the same framework, each individual was assigned to a biogeographic province as well as an ecoregion (cf. Fig. 1B and C) based on the location from which it was sampled. Seventeen of the biogeographic provinces in the Udvardy model and 50 ecoregions in the Olson model were represented by samples in our CR data set (Table S3), accounting for 100% and 81% of the provinces and ecoregions respectively in which bushbuck are known to occur. The remaining unsampled ecoregions are small, primarily montane or highly isolated. Overall model strength was assessed by one-group AMOVA (Table 1). The variance among populations (*F*_ST_) provided a measure of the maximum genetic variation accounted for by each model. The same data set was used for each test, thus *F*_ST_ was used to assess each model relative to one based on terminal haplogroup definition (the molecular model), which is expected to return the upper limit of *F*_ST_ for the data set.

Model testing was also performed by multivariate matrix regression (MMR), with the software DISTLM [51]. While this does not allow for the hierarchical partitioning of variance, the advantage of this method over AMOVA is that it uses an explicit linear model and does not require an *a priori* user-defined population structure. Furthermore, the geographic distance separating sampling locations of widely distributed mammal species may significantly influence genetic structure [52], [53]. MMR allows the quantification of this influence, conditional on that of biogeography and phenotype. In addition, the forward selection method [DISTLM *forward*, 54] sequentially determines which of two sets of variables (geography *versus* phenotype, biogeography or molecular) fit the data best, and the proportion of the remaining variance described by the secondary set of variables. Pair-wise genetic distances between all 239 sampling locations was used as the response matrix and tested against phenotypic, biogeographic, molecular and geographic predictor matrices. A matrix of latitude and longitude covariables for all sampling locations was used to assess the conditional and sequential influence of geographic distance on the models being tested.

In a species exhibiting such marked local differentiation, many of the haplogroups defined by monophyly may be expected to exclusively inhabit the ecoregions to which they have become associated. Ecoregions inhabited by more than one haplogroup are also expected to occur, first due to inadequacies in the underlying biogeographic model and second, due to the general ubiquity of this species. We addressed the former by refining our biogeographic model and analysed the latter by defining areas of core habitation for each haplogroup. The nature of the core ecoregions linked by a single haplogroup allowed the inference of ecological affinities as well as the identification core biomes (Fig. 3).

## Supporting Information

Table S1 Classification and reference details of 485 bushbuck specimens
(0.13 MB XLS)

Table S2 Summary statistics for higher, intermediate and terminal level haplogroups as defined in Figure 1
(0.02 MB XLS)

Table S3 Refining the Olson model and defining core ecoregions for 23 bushbuck haplogroups.
(0.03 MB XLS)
